# Rare, epilepsy-related disorder including intellectual disability – A scoping review of caregivers’ identified information needs

**DOI:** 10.1177/17446295211002348

**Published:** 2021-05-17

**Authors:** Merete Kristin Tschamper, Silje Systad

**Affiliations:** Oslo University Hospital, Norway; Oslo University Hospital, Norway

**Keywords:** caregiver, epilepsy, information need, intellectual disability, rare

## Abstract

Persons with rare, epilepsy-related disorders often have intellectual disability and need long-term care. Informal and formal caregivers need information in order to care for the persons in a safe way.

**Aims:**

The aims of this review were: (1) to obtain an overview of caregiver-reported information needs; and (2) to investigate if there are information needs that are unique for caregivers of persons with rare epilepsies.

**Method:**

We followed the scoping review framework outlined by Arksey and O’Malley and the preferred reporting items outlined by PRISMA.

**Results:**

Among the 17 articles that met the inclusion criteria, 5 included caregivers of persons with rare epilepsies. Categories of information needs: (1) Medical information; (2) Information on how to cope with emotional distress; (3) Experiential information from peers; and (4) Interdisciplinary information exchange. The need for disorder-specific information seemed particularly important for caregivers of persons with rare epilepsies.

**Conclusion:**

There is a need for further studies, particularly on formal caregivers’ information needs.

## Background

Rare, epilepsy-related disorders manifest with heterogeneous symptoms and are associated with early onset epilepsy, which is difficult to treat when presenting alongside intellectual disability (Scheffer et al., 2017). The disorders are often complex conditions that require lifelong treatment and care support (Van der Zeijden and Huizer, 2010). Psychosocial problems appear frequently, along with movement difficulties and other syndrome-specific physical symptoms (McTague and Cross, 2013). The symptoms sometimes change across age (Both et al., 2018). Persons with intellectual disability may have a reduced capacity to communicate discomfort and may rely on informal and formal caregivers to respond to their everyday needs, to ensure adherence to treatment along with the safe handling of seizures (Both et al., 2018; Brinciotti and Matricardi, 2019; Camfield and Camfield, 2014; McTague and Cross, 2013). The informal and formal caregivers responsible for persons with such complex conditions need information in order to ensure optimal treatment and care (Camfield and Camfield, 2014; Jensen et al., 2017a). In this review, the term “informal caregiver” and “formal caregiver” refer to employment status. An “informal caregiver” is typically not employed (for example, a parent), while a “formal caregiver” is employed and may or may not have a formal health education.

The caregivers’ need for comprehensive information has been acknowledged at *national, organisational* and *individual* levels through legal regulations and clinical guideline recommendations from, for example, the National Institute for Health Care Excellence (NICE) and the Scottish Intercollegiate Guidelines Network (SIGN) (National Institute for Health and Care Excellence, 2012; Scottish Intercollegiate Guidelines Network, 2015). These guidelines emphasise that such information should be comprehensive and tailored to the resources, time of diagnosis of a rare, epilepsy-related disorder and phase of life of the individual (National Institute for Health and Care Excellence, 2012; Scottish Intercollegiate Guidelines Network, 2015).

Despite legal rights and clinical guideline recommendations, the “Consensus guidelines into management of epilepsy in adults with an intellectual disability” (Kerr et al., 2009) described few studies of high evidence to support evidence-based management of epilepsy in adults with intellectual disability. According to Kerr et al. (2009), some caregivers might not be offered sufficient information by health professionals to provide adequate support to the persons they care for (Kerr et al., 2009).

This scoping review aimed to obtain an overview of research regarding the need for information reported by the informal and formal caregivers of persons with rare epilepsies. It also aimed to explore if the information needs identified by caregivers of persons with rare, epilepsy-related disorders differed from those of caregivers of persons with other epilepsies alongside intellectual disability.

## Method/data collection

In order to identify relevant research, we followed five of the six (optional) stages of the scoping review framework outlined by Arksey and O’Malley (2005) and the PRISMA Extension for scoping reviews (PRISMA-ScR): Checklist and explanation (Arksey and O’Malley, 2005; Munn et al., 2018; Tricco et al., 2018). Researchers R1 (MKT) and R2 (SS), professionals with clinical experience working with persons with rare, epilepsy-related disorders and their informal and formal (community) caregivers, developed a structured search protocol for the review.

### Stage 1: Identifying the research question

The research questions for this review were developed throughout the research process as we (R1 and R2) became familiar with the available literature. Due to the low prevalence of rare, epilepsy-related disorders, we expected to find a limited amount of primary research on the subject (von der Lippe et al., 2017). This expectation was confirmed by pilot searches in the Medline and Embase databases. In order to prevent overlooking relevant information, we chose broad inclusion criteria to obtain an overview of existing research on information needs identified by caregivers (Arksey and O’Malley, 2005).

### Stage 2: Identifying relevant studies

A librarian performed a broad search with the following search categories: clinical population/diagnostic terms, outcomes, type of research/methodological terms, language, full text available, and publication time. The Medline, PsycInfo, Cinahl, and Embase databases were chosen, as they cover a wide range of health research in Europe, the USA, and Australia written in the English and Scandinavian languages. The continents and countries mentioned have similar health services to our own, and the research from those countries is supposed to be comparable to our own health services. The search was merged using EndNoteX9. After removing duplicates and limiting the publication time of the studies to the years 2000–2019, 3900 articles were identified (see Online Appendix 1).

### Stage 3: Study selection

The researchers (R1 and R2) screened the 3900 articles by title and abstract against the inclusion criteria presented in Table 1. To increase consistency, the first 200 publications were screened by each researcher independently. The articles included by each researcher (R1 and R2) were then compared and showed a high degree of agreement. Any differences concerning the articles included were discussed until a consensus was agreed upon. Because of the high degree of agreement between R1 and R2, it was assumed that the inclusion and exclusion criteria gave a clear direction for the selection of articles. The remaining 3700 articles were then shared between R1 and R2.

**Table 1.** Inclusion and exclusion criteria.

	**Inclusion criteria**	**Exclusion criteria**
**Clinical population**	Informal and formal caregivers to persons with intellectual disability and epilepsy	Informal and formal caregivers to persons without intellectual disability and epilepsy Patients
**Outcomes**	Descriptions of caregivers (informal and formal) information needs	No descriptions of caregivers (informal and formal) information needs
**Databases**	Medline (Ovid), Cinahl, Embase (Ovid), PsycInfo (Ovid)	Other databases
**Type of research**	Peer reviewed primary qualitative, quantitative and multi method studies, full text available	Reviews, conference annotations and unpublished work, full text not available
**Language**	English and Scandinavian	Other languages
**Publication time**	Between 2000 and 2019	Before 2000

After screening by title and abstract, 50 articles were included and screened on by full text. Additionally, 14 articles were excluded as the caregivers were not the study population but participated on behalf of their children. Of the remaining 36 articles, R1 found that 19 articles did not report caregiver information needs. This was verified by R2, and they were excluded.

**Figure 1.**
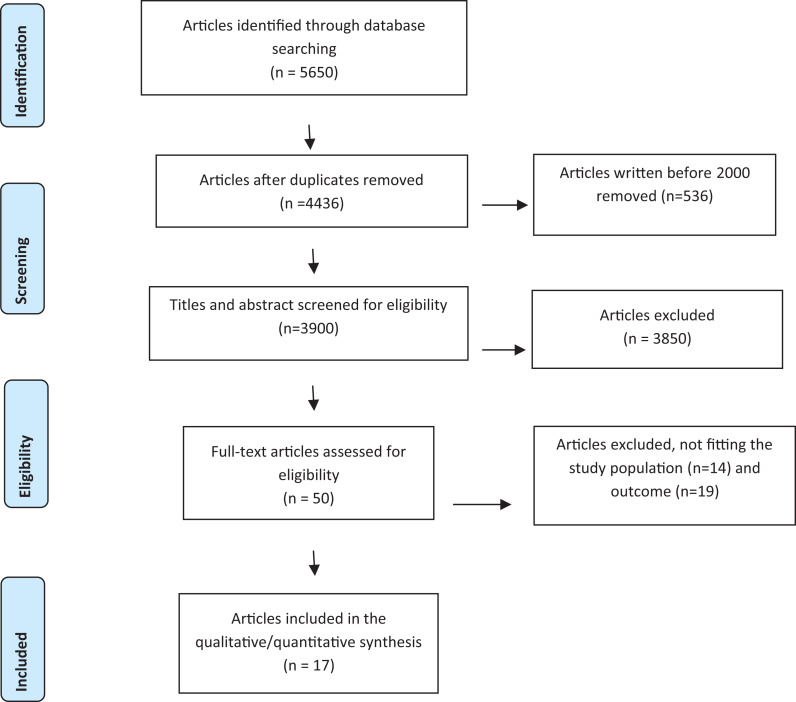
PRISMA 2009 flow diagram.

After full text screening, 17 articles remained for inclusion. The study selection process is presented in a PRISMA flow chart (Figure 1).

### Stage 4: Charting the data

In order to chart the data in a standardised manner, R1 documented the descriptive data of each article, including the author, year, country of origin, study population, patients’ diagnoses (epilepsy and level of intellectual disability), sample size, study design/methods, main findings, limitations, and information needs.

#### Charting the identified information needs

The process of identifying and categorising the information needs reported by the caregivers was challenging, as none of the articles had the caregivers’ information needs as a main focus. Thus, the articles needed to be scanned thoroughly for such information. The process of categorising the information included reading through each article and highlighting the caregiver-reported information needs. The information needs were categorised thematically, inspired by thematic analysis as initiated by Clark and Braun (2012). R1 scanned the articles, highlighted the parts that described the caregivers’ need for information, and created categories that defined the different information needs. After identifying the categories, R1 extracted and charted the data from the articles in an Excel form.

The charting of information was a challenging process, as many of the information needs could fit into more than one category and seemed to be interconnected. For example, the needs for medical information and interdisciplinary information exchange were defined as crucial in the transition to adulthood; the formal caregivers’ lack of person-centred, medical information in respite care increased the informal caregivers’ level of experienced stress, and thus the need for information on how to cope with emotional stress and so on. Therefore, some of the originally chosen categories were merged. R1 then reread the articles to check if the information described in the original articles had been changed in the chosen categories. Before deciding the categories of information, the categories were discussed with R2.

### Stage 5: Collating, summarising and reporting the results

In the fifth and final stage of the review process, we summarised the extracted data. At the end of each category we comment on the information identified by caregivers of persons with rare epilepsies.

## Results

### Study characteristics

#### Article publication information

An overview of the study characteristics is presented in Online Appendix 2. Out of 3900 articles, 17 met the inclusion criteria. The articles were published between 2006 and June 30, 2019, and the majority (n = 14) were published between 2011 and 2019. The studies were conducted in Europe (Netherlands n = 2, France n = 2, UK n = 4, UK/Ireland n = 2), America (USA n = 2, Canada n = 3, Brazil n = 1), and Australia n = 1. The study designs included qualitative studies, interviews n = 5, interview/focus group n = 1, participative action research n = 1, quantitative studies, surveys n = 8, longitudinal/structured phone interviews n = 1, and mixed methods of register data and interviews n = 1.

#### Study population

Informal caregivers (family, parent, parent proxy) were the study population in 16 of the 17 studies, either alone (n = 6), together with the person/patient (n = 7), or together with the formal caregiver (n = 3). Formal caregivers (professionals and managers in respite care) were the study population in one article (n = 1).

#### The person’s diagnosis, level of intellectual disability and age

Caregivers of persons with rare, epilepsy-related disorders and intellectual disability were included in five articles: Dravet Syndrome (n = 2) Dravet Syndrome/Lennox-Gastaut syndrome (n = 1), and Tuberous sclerosis complex (n = 2). The remaining 12 articles included caregivers of persons with epilepsy and intellectual disability. Only two of the articles specify the person’s type of epileptic seizures.

The person’s level of intellectual disability was specified in six articles, ranging from mild to profound according to the International Classification of Diseases and related health problems (ICD-10) system of classification of ‘mental retardation’ (National Center for Health Statistics, 1992).

Of the articles in which the age of the person being cared for was specified 10 included adults 18 years or older, while 3 included children and young adults (younger than 24 years old).

### Information needs

The charting process identified four categories of information needs in the review: (1) Medical information; (2) Information on how to cope with emotional distress; (3) Experiential information from peers; and (4) Interdisciplinary information exchange. Categories 1 to 3 describe *the content* of the information the caregivers need, whereas category 4 describes with *whom* this information needs to be exchanged.

Information needs that seemed to be unique for the caregivers of persons with rare epilepsies are described at the end of each category.

### Medical information

The need for medical information concerning the individual’s diseases, symptoms and treatment options was the most frequently reported information need in this review (Bellon et al., 2014; Both et al., 2018; Buelow et al., 2006; Deepak et al., 2012; Desnous et al., 2011; Gordon et al., 2014; Jensen et al., 2017b; Kehyayan and Hirdes, 2018, 2019; McGrother et al., 2006; Nolan et al., 2008; Schultz, 2013; Thompson et al., 2013, 2014; Vallenga et al., 2008). Both the informal and formal caregivers requested information on how the two diagnoses, epilepsy and intellectual disability (diagnosis, treatment options, prognosis) would interact and affect the overall prognosis (Both et al., 2018; Kehyayan and Hirdes, 2018, 2019; McGrother et al., 2006; Thompson et al., 2013, 2014).

In the majority of the studies, information regarding side effects of prescribed medication and seizure-handling skills, was emphasised as crucial to ensure the safe handling of seizures and acute medicine (Bellon et al., 2014; Both et al., 2018; Buelow et al., 2006; Deepak et al., 2012; Desnous et al., 2011; Jensen et al., 2017b; Kehyayan and Hirdes, 2018, 2019; Nolan et al., 2008; Schultz, 2013; Thompson et al., 2013, 2014; Vallenga et al., 2008). Both informal and formal caregivers stressed the need to write an emergency protocol with clear instructions on how to handle seizures, including those that do not stop despite the use of acute medicine, status epilepticus (SE) (Deepak et al., 2012; Desnous et al., 2011; Nolan et al., 2008; Thompson et al., 2013; Vallenga et al., 2008). The approach should include information and actions taken to avoid harm from falling during seizures. Finally, the caregivers emphasised information on contextual and personal factors that may trigger seizures in order to prevent seizure (Vallenga et al., 2008).

Regarding rare epilepsies, only the particular need for information about fever as a seizure trigger in Dravet syndrome differed from the medical information needs mentioned by caregivers of persons with other epilepsies (Desnous et al., 2011; Jensen et al., 2017b; Nolan et al., 2008).

### Information on how to cope with emotional distress

Several informal caregivers reported the need for information on strategies on how to cope with stress in order to manage the challenges of caring for a person with a severe and, for some, potentially life-threatening condition (Desnous et al., 2011; Jensen et al., 2017b; Kehyayan and Hirdes, 2018, 2019; Nolan et al., 2008; Walker et al., 2014). Some informal caregivers described the feeling of always being “on guard, waiting for the next seizure”, as stressful (Desnous et al., 2011; Jensen et al., 2017b; Kehyayan and Hirdes, 2018, 2019; Nolan et al., 2008). Some suggested that this emotional stress was amplified by formal caregivers involved with the person who have insufficient seizure-handling information (Kehyayan and Hirdes, 2019; Thompson et al., 2014). Several expressed a reluctance to leave the person in respite care, in the care of other family members, or even in school because they feared for the person’s safety due to inadequate caregiver competence (Desnous et al., 2011; Jensen et al., 2017b; Nolan et al., 2008; Thompson et al., 2014).

In the study by Thomsen et al. (2014), informal caregivers reported that information on how to cope emotionally with the burden of caring for the person was rarely an issue discussed in consultations with professionals (Thompson et al., 2014).

Emotional distress and anxiety caused by the responsibility of ensuring safety for their loved one was emphasised in all the articles concerning caregivers of persons with Dravet syndrome and Lennox-Gastaut syndrome (Desnous et al., 2011; Jensen et al., 2017b; Nolan et al., 2008).

### Experiential information from peers

Several informal caregivers described the need for experiential information from peers on how to find practical solutions to everyday problems (Jensen et al., 2017b; Nolan et al., 2008; Schultz, 2013). Through peers, they received crucial information on how to care for their own health while facing sleep deprivations, social isolation, how to handle strain put on siblings, and reduced time with their spouse (Kehyayan and Hirdes, 2018; Thompson et al., 2014). Possible solutions to meet their own health needs included information on coping strategies as well as information regarding their legal rights to care support (Kehyayan and Hirdes, 2018). In the study by Thomsen et al. (2013), some informal caregivers described information from peers through the Internet as their main source of medical information concerning the person’s diagnosis (Thompson et al., 2013).

Several informal caregivers of persons with Dravet syndrome and Lennox-Gastaut syndrome reported that talking to someone in a similar situation reduced the emotional burden of care (Jensen et al., 2017b; Nolan et al., 2008). Meeting physically was experienced as difficult due to the unpredictable occurrence of seizures, along with sleep and behaviour problems (Jensen et al., 2017b; Nolan et al., 2008). Some informal caregivers suggested that flexible digital ways to “meet” peers were possible solutions to the challenge of finding time for face-to-face meetings (Jensen et al., 2017b).

### Interdisciplinary information exchange

Some informal caregivers described the fragmentation of services and discontinuity of providers in adult service as stressful for the persons they cared for as well as themselves (Bar et al., 2019; Both et al., 2018). The need for information exchange between the multidisciplinary providers involved in the follow-up was emphasised in 15 articles (Baca et al., 2018; Bar et al., 2019; Bellon et al., 2014; Both et al., 2018; Buelow et al., 2006; Deepak et al., 2012; Jensen et al., 2017b; Kehyayan and Hirdes, 2018, 2019; McGrother et al., 2006; Nolan et al., 2008; Schultz, 2013; Thompson et al., 2013, 2014; Vallenga et al., 2008). Insufficient information leading to inadequate treatment of coexisting difficulties, such as behavioural problems or psychological symptoms, was mentioned in several studies (Both et al., 2018; Buelow et al., 2006; Kehyayan and Hirdes, 2018, 2019; McGrother et al., 2006; Thompson et al., 2013).

Some informal and formal caregivers emphasised the need to involve informal caregivers when interdisciplinary information was exchanged to ensure that person-centred information was taken into account (Bar et al., 2019; Bellon et al., 2014; Both et al., 2018; Buelow et al., 2006; Deepak et al., 2012; Jensen et al., 2017b; Kehyayan and Hirdes, 2018, 2019; McGrother et al., 2006; Thompson et al., 2013). Additionally, the involvement of informal caregivers was considered important in order to initiate shared decision-making in questions regarding treatment (Bellon et al., 2014; Thompson et al., 2013; Vallenga et al., 2008). The transition from paediatric care to adult care was described as a particularly vulnerable time due to discontinuity of care caused by inadequate information transfer (Baca et al., 2018; Bar et al., 2019; Both et al., 2018; Buelow et al., 2006). The caregivers also emphasised the need for repeated comprehensive evaluation of psychological, physiological, social, socioeconomical and environmental factors influencing the person’s quality of life (Baca et al., 2018; Bar et al., 2019; Both et al., 2018).

The need for interdisciplinary information exchange in the transition to adulthood was the focus in the two articles including caregivers of persons with the rare epilepsy-related diagnosis tuberous sclerosis complex (Bar et al., 2019; Both et al., 2018). The informal caregivers request easily accessible professionals that can distribute treatment information to ensure a safe handling of the person (Bar et al., 2019; Both et al., 2018). The caregivers feared that the lack of continuity of care in adult health services increases the risk of insufficient treatment due to the rare nature and limited public knowledge of the disorders (Bar et al., 2019; Both et al., 2018).

## Discussion

This scoping review aimed to provide an overview of research regarding the need for information reported by the informal and formal caregivers of persons with rare epilepsies, and to explore if the information needs identified by these caregivers differed from those of caregivers of persons with other epilepsies including intellectual disability.

We found that the articles described what the caregivers need to know and with whom this information needs to be exchanged. Since 14 of the 17 articles have been published after 2011, and thus are less than 10 years old, we can assume that the information needs described are relevant in the current situation. However, the review revealed a striking lack of research regarding caregivers’, in particularly formal caregivers’, need for information in order to care for persons with rare epilepsies including intellectual disability in a safe way.

### What do the caregivers need to know?

In this review, the reported information needs were largely focused on what the informal caregivers *needed the formal caregivers to know* in order to care for the person. Several informal caregivers in this review reported that systematic training of the informal caregivers was not properly organised (Both et al., 2018; Deepak et al., 2012; Thompson et al., 2013, 2014; Vallenga et al., 2008). The consequences of being left with caregivers who have inadequate training are potentially fatal for the child. The NICE (2012) guidelines recommend that all relevant caregivers should receive training to be able to administer acute medicine according to a specified protocol (National Institute for Health and Care Excellence, 2012). Distrust in the competence of formal caregivers could mean additional psychological distress for the informal caregivers. In addition, it may keep them from leaving their loved ones in respite care and thus reduce the amount of time they are relieved from the caregiver burden, increasing the risk of being worn out. Mandatory routines are urgently needed to ensure the NICE (2012) recommendations are followed (National Institute for Health and Care Excellence, 2012).

The emotional burden on informal caregivers of persons with chronic diseases found in this review has been described in other recent studies (Khangura et al., 2015; Pelentsov et al., 2015). The emotional distress includes dealing with emotions, such as shock, anger, distress, fear, denial, guilt and the feeling of being constantly “on guard, waiting for the next seizure” (Khangura et al., 2015; Pelentsov et al., 2015). A recent scoping review by Pelentshov et al. (2015) suggested that exchanging information in peer-support groups with persons who share a similar situation reduced the emotional burden of caring for a person with a severe, chronic condition. In addition, sharing information and receiving practical guidance and emotional support made the informal caregivers feel less alone (Pelentsov et al., 2015).

In this study (Pelentsov et al., 2015), the informal caregivers found it difficult to meet physically, and digital platforms gave them an arena to meet others who experienced similar challenges. The growth of social networking has facilitated the sharing of information and worries through peer-support groups, personal blogs, twitter chats and more. However, as epilepsy and intellectual disability manifest heterogeneously, the effect of medical treatment and the resulting side effects will vary between individuals. Thus, there is a risk of peers in non-professional-led support groups spreading misinformation, leading to non-adherence to treatment (Niela-Vilén et al., 2014). Medical information should be discussed with the doctor in charge of the treatment before action is taken. However, some informal caregivers suggested that the need for information on how to manage the emotional burden of caring for a person with epilepsy was not met in consultations with health professionals. Striving for open communication during clinical encounters on how peer-support groups can be beneficial for informal caregivers may be important to support the caregivers. Studies on the beneficial outcomes of professionally led peer-support groups for informal caregivers are promising but inconclusive in terms of caregiver outcomes (Niela-Vilén et al., 2014; Pomery et al., 2016).

### Efficient communication pathways for multidisciplinary information exchange

Despite the NICE (2012) and SIGN (2015) clinical guideline recommendations regarding comprehensive treatment, the caregivers in this review reported a lack of efficient communication pathways and arenas to exchange multidisciplinary information (National Institute for Health and Care Excellence, 2012; Scottish Intercollegiate Guidelines Network, 2015). A recent review by Robertson et al. (2017) described the need for studies on how to organise health services more efficiently to ensure that multidisciplinary providers involved in care received sufficient information to safeguard persons with epilepsy and intellectual disability (Robertson et al., 2017).

Informal caregivers in this study indicated that their information about the person’s support needs was not requested when multidisciplinary information was exchanged, posing a risk of reduced shared decision-making (Bellon et al., 2014; Vallenga et al., 2008). Shared decision-making involves defining the person’s health problems, resources and presenting treatment options (Lin et al., 2018). When informal caregivers’ information about the person’s support needs are not requested or incorporated in the person’s care plan, it may pose a risk of reduced person-centred treatment. Including the informal caregivers in the process of shared decision-making may increase user involvement in treatment and improve the quality of health services (Lin et al., 2018). A recent study (Lin et al., 2018) documented that shared decision-making was less common for patients with complex needs than for those without complex needs. The need for mandatory routines to ensure shared decision-making for adults with epilepsy and intellectual disability has been emphasised in several studies (Kerr et al., 2009; Lin et al., 2018; Vallenga et al., 2008).

The caregivers in this study reported the transition to adulthood as *the time* in the person’s lifespan when the need for person-centred multidisciplinary information exchange was particularly important to ensure adequate treatment of the person (Baca et al., 2018; Bar et al., 2019; Both et al., 2018). A Cochrane review (Rachas et al., 2016) emphasised the importance of initiating lifelong relationships between persons with childhood-onset chronic conditions and informed formal caregivers to ensure continuity of services and a safe transition (Rachas et al., 2016). There are a variety of transition programmes, but as each person’s symptoms and needs are individual, a transitional programme may be hard to standardise. There is a need for further research on the efficiency of such programmes (Camfield et al., 2019).

Notably, the NICE (2012) and SIGN (2015) guideline recommendations highlight that epilepsy in old age poses unique challenges, such as increased risk of comorbidity and polypharmacy, and increased vulnerability to adverse drug reactions (National Institute for Health and Care Excellence, 2012; Scottish Intercollegiate Guidelines Network, 2015). Further, the need for new repeated screenings and assessments to adapt treatment is recommended (National Institute for Health and Care Excellence, 2012; Scottish Intercollegiate Guidelines Network, 2015). This suggests a specific need for caregiver information in the transition from adulthood into old age. However, we were not able to find research on specific caregiver information needs when the person is in the transition to old age.

### Do rare, epilepsy-related disorders require different caregiver information?

The three articles concerning caregivers of persons with Dravet syndrome (Desnous et al., 2011; Jensen et al., 2017b; Nolan et al., 2008), as well as the one regarding Lennox–Gastaut syndrome (Jensen et al., 2017b), all reported specific information needs regarding how to handle attacks of fever to prevent seizures, and how to administer acute medicine to stop seizures to prevent SE (Desnous et al., 2011; Jensen et al., 2017b; Nolan et al., 2008). The specific medical focus in the articles was not surprising, as fever is known to be seizure provoking, particularly in Dravet syndrome. In addition, people with Dravet syndrome or Lennox–Gastaut syndrome have an increased risk of prolonged seizure leading to SE compared with the general epilepsy population (Dravet, 2016; Gibson, 2014). Untreated, convulsive SE can be life-threatening, and the condition requires emergency treatment by trained medical personnel in a hospital setting (Epilepsy Foundation, 2019). Meanwhile, prolonged seizures and SE are more common in people with intellectual disability than in the general population, and are not unique to persons with rare, epilepsy-related disorders including intellectual disability (Robertson et al., 2015). A systematic review by Robertson et al. (2015) concluded that epilepsy and the risk of SE increases with increasing levels of intellectual disability, and services must be equipped with the skills and information needed to manage SE (Robertson et al., 2015). Either way, to care for a person with a potentially life-threatening condition may naturally cause emotional distress. The findings suggested that information on how to cope with this emotional burden was rarely addressed in consultations with professionals. As a result, the caregivers may seek information and support from other resources, such as peer-support groups.

This review identified two articles concerning caregivers of persons with tuberous sclerosis complex, both focusing on treatment challenges in the transition to adulthood (Bar et al., 2019; Both et al., 2018). Tuberous sclerosis complex is a genetic disease associated with tubers (benign tumours) that may affect various organs, including the cortex, heart, lungs, kidneys and skin, leading to various symptoms that require medical treatment. The person needs to follow recommended regularly screenings of the affected organs and to receive treatment by specialists (Bar et al., 2019; Both et al., 2018; Krueger et al., 2013). Without these regular screenings, the person may experience severe health consequences, such as kidney failure and hydrocephalus (Krueger et al., 2013). Research has shown that knowledge about rare diseases is sparse, not only in the general public but also among health care providers (Rodwell and Aymé, 2015). Persons with tuberous sclerosis complex may be particularly likely to receive inadequate treatment as a consequence of discontinuity of services in the transition to adulthood due to the rare nature of the condition. However, the need for multidisciplinary, person-centred information in the transition to adulthood was also reported by caregivers of persons with epilepsy and intellectual disability and is thus not unique to persons with rare, epilepsy-related disorders (Baca et al., 2018; Bar et al., 2019; Both et al., 2018).

### Strengths and limitations – Implications for further research

The structured methodological process implemented in this scoping review is a strength that increases the validity of the findings (Arksey and O’Malley, 2005; Tricco et al., 2018). The study population in the articles described similar needs for caregiver information and the challenges in receiving adequate information, which might indicate directions for further studies.

The findings were naturally skewed towards the informal caregivers’ information needs, as 16 of the 17 articles included informal caregivers as the study population. Because the review included a limited number of articles, and mainly informal caregivers as the study population, the results need to be interpreted with caution.

There were no articles in this study from Asian or African countries. The findings in this study may therefore not be representative of caregivers from those continents, as cultural factors and medical systems may influence the information needs.

The impact of the level of intellectual disability and the epileptic seizure type on the caregivers’ needs for information was not discussed in the articles included in this review. Age was also not discussed. As the level of intellectual disability, age and the severity of seizures are known to have an impact on a person’s self-management and need for care support, these are areas that require further research (Robertson et al., 2017).

This review only identified five articles concerning three different rare, epilepsy-related disorders. However, there are several identified rare epilepsies, and so the reported information needs may not be representative of other rare epilepsy-related diagnosis (Nariai et al., 2018; Wang et al., 2017).

## Conclusion

Despite national clinical recommendations, the caregivers in this review described a lack of efficient communication pathways and areas to exchange multidisciplinary information between the providers involved in the follow-up. There seem to be an urgent need for studies on how to organise health services more efficiently in order to ensure that formal caregivers receive sufficient information to safeguard persons with epilepsy and intellectual disability. The findings also indicated that the need for information on coping strategies to manage the caregiver burden was unmet and should be further explored.

Diagnosis-specific medical information seemed particularly crucial for the caregivers of persons with rare, epilepsy-related disorders. Moreover, this need appeared to be related to the fact that public knowledge of the rare disorders is limited, and there was a risk of severe health consequences for the person when caregivers were not sufficiently informed about the symptoms. There were limited relevant studies, and thus there is a need for further research on the information needs of caregivers of persons with rare, epilepsy-related disorders in general and of formal caregivers in particular.

## Supplemental material

Supplemental Material, sj-pdf-1-jld-10.1177_17446295211002348 - Rare, epilepsy-related disorder including intellectual disability – A scoping review of caregivers’ identified information needs Click here for additional data file. Supplemental Material, sj-pdf-1-jld-10.1177_17446295211002348 for Rare, epilepsy-related disorder including intellectual disability – A scoping review of caregivers’ identified information needs by Merete Kristin Tschamper and Silje Systad in Journal of Intellectual Disabilities

Supplemental Material, sj-pdf-2-jld-10.1177_17446295211002348 - Rare, epilepsy-related disorder including intellectual disability – A scoping review of caregivers’ identified information needs Click here for additional data file. Supplemental Material, sj-pdf-2-jld-10.1177_17446295211002348 for Rare, epilepsy-related disorder including intellectual disability – A scoping review of caregivers’ identified information needs by Merete Kristin Tschamper and Silje Systad in Journal of Intellectual Disabilities
