# Supplementary material for: Rare, epilepsy-related disorder including intellectual disability – A scoping review of caregivers’ identified information needs
Source: J Intellect Disabil. 2021 May 17;26(3):704–17. doi: 10.1177/17446295211002348 (PMC9442773; doi:10.1177/17446295211002348)
Supplement: Supplemental Material, sj-pdf-1-jld-10.1177_17446295211002348 - Rare, epilepsy-related disorder including intellectual disability – A scoping review of caregivers’ identified information needs [file sj-pdf-1-jld-10.1177_17446295211002348.pdf]

Appendix 1.

Documentation of literature search

Documentation on the literature search for *Caregivers (professional/family/unformal) need for health information in order to take care of persons with rare, epileptic conditions, including intellectual disability.*

The following databases were searched:

Database	Number of retrieved references
Medline (Ovid):	877
PsycInfo (Ovid):	2644
Embase (Ovid):	396
CINAHL (Ebsco):	1604
Cochrane Library:	129
Number of references before deduplication:	5650
Number of references after deduplication:	4436

Some comments:
All references are reviewed and duplicates are removed, though some may have been missed. In cases of references to Cochrane Systematic Reviews with same title and CD-number, the oldest are deleted as it is withdrawn.

In cases of references with the same title and authors, but different journals, these are not removed even though they ofhten are duplicate publications (same article in different journals), or they are a conference contributions and the publicated article.

Titles within [square brackets] = article in a non-English language.

EndNote
References are imported to an EndNote Library named:
Rare epileptic diagnosis information_aug19.enl

To save the EndNote Library:
Double click the EndNote file, it opens and can be saved to your computer. Click File -> Save as, and save to your computer.

Search syntax:

Ovid-databases:

/	After an index term indicates a subject heading were selected.
.tw.	Indicates a search for a term in title or abstract
.kw.	= keyword heading
.kf.	= keyword heading word
*	At the end of a term indicates that this term has been truncated.
Adj3	Indicates a search for two terms next to each other, in any order, up to 3 words in between.

CINAHL/ EBSCO

MH	Search for subject heading
TI	Search for word in title
AB	Search for word in abstract
*	At the end of a term indicates that this term has been truncated.
N3	Indicates a search for two terms next to each other, in any order, up to 3 words in between.

Cochrane library

ti,ab,kw	Indicates search for word in title, abstract or keyword
NEAR/3	Indicates a search for two terms next to each other, in any order, up to 3 words in between.

The Medline search is structured as follows (searches in other databases are adapted from this):

**Topic**	**Search line**
**Patients:**	
Epilepsy with intellectual disabilities	1-4
Specific epilepsy diagnoses	5-18
All Patients	20
Families and next of kin	21-24
Caregivers or health staff at residential facilities	25
All families and health staff	26
Experiences, Information, knowledge etc	27-34
Patients, families and information	35

Search is not limited by year, language or research design.

Embase, Medline and PsycInfo

Embase Classic+Embase 1947 to 2019 August 14,

Ovid MEDLINE(R) ALL 1946 to August 14, 2019,

PsycINFO 1806 to August Week 2 2019

Date of search: August 16, 2019

Results: Medline 877, EMBASE 2644, PsycInfo 396.

1	exp Epilepsy/	381663
2	(epilep* or seizure* or convulsion*).tw,kw,kf.	560696
3	Intellectual Disability/ or ((mental or Intellectual or cognitive) and (disabilit* or retardation or Deficiencies or Deficiency or impairment)).tw,kw,kf.	531859
4	(1 or 2) and 3	38784
5	Dravet syndrome.tw,kw,kf.	2502
6	(GLUT1 deficiency syndrome or glucose transporter type 1 deficiency syndrome).tw,kw,kf.	526
7	Lafora Disease/ or lafora.tw,kw,kf.	7033
8	TSC1.tw,kw,kf.	4554
9	TSC2.tw,kw,kf.	5773
10	Landau-Kleffner Syndrome/ or Landau Kleffner.tw,kw,kf.	1735
11	Lennox Gastaut Syndrome/ or Lennox Gastaut.tw,kw,kf.	5728
12	exp Epilepsies, Myoclonic/	11129
13	(myoclonic epilep* or myoclonic encephalopath*).tw,kw,kf.	7878

14	Unverricht-Lundborg Syndrome/ or unverricht-lundborg.tw,kw,kf.	6991
15	Sturge-Weber Syndrome/ or Sturge Weber.tw,kw,kf.	4795
16	Ohtahara.tw,kw,kf.	605
17	Aicardi Syndrome/ or Aicardi.tw,kw,kf.	2008
18	GRIN1.tw,kw,kf.	593
19	or/4-18	73127
20	exp Family/	1127632
21	"Child of Impaired Parents"/	5407
22	Caregivers/	111760
23	(parent* or mother* or father* or daughter* or son* or sibling* or sister* or brother* or wife* or spouse* or husband* or partner* or meaningful person* or family member* or stepfamil* or next-of-kin or kinship or relatives or caregiver* or carer or carers or dependent* or significant others).tw,kw,kf.	6410063
24	(exp Health Personnel/ or (staff or nurse or nurses or Personnel).tw,kw,kf.) and (exp Residential Facilities/ or Community Health Services/ or (Community Health Service* or home or homes or Residential Facilit* or living facilit*).tw,kw,kf.)	158860
25	or/20-24	6901826
26	Life Change Events/	48177
27	(life adj3 (experience or events or event or transition or transitions or transitional or change or changes)).tw,kw,kf.	113599
28	quality of life/ or (life adj3 quality).tw,kw,kf.	936606
29	((self adj3 (management or managing)) or adherence or compliance or coping or resilience).tw,kw,kf.	897184
30	(knowledge or information or education or experience).tw.	7081244
31	knowledge/	46794
32	(need or needs).tw,kw,kf.	3013033
33	or/26-32	10502693

34	19 and 25 and 33	3049
35	exp epilepsy/	381663
36	exp seizures/	228870
37	(epilep* or seizure* or convulsion*).tw.	551612
38	exp Intellectual Development Disorder/ or exp Cognitive Impairment/ or ((mental or Intellectual or cognitive) and (Disability or retardation or Deficiencies or Deficiency or cognitive impairment)).tw.	872194
39	(35 or 36 or 37) and 38	60038
40	lennox gastaut syndrome/	4026
41	(Dravet syndrome or TSC1 or TSC2 or Landau Kleffner or Lennox Gastaut or myoclonic encephalopath* or Sturge Weber or Ohtahara or Aicardi or GRIN1 or (GLUT1 deficiency syndrome or glucose transporter type 1 deficiency syndrome) or lafora or unverricht-lundborg).tw.	23679
42	or/39-41	82448
43	exp Family/	1127632
44	exp parents/	490170
45	exp parenting/	207022
46	caregivers/	111760
47	(parent* or mother* or father* or daughter* or son* or sibling* or sister* or brother* or wife* or spouse* or husband* or partner* or meaningful person* or family member* or stepfamil* or next-of-kin or kinship or relatives or caregiver* or carer or carers or dependent* or significant others).tw.	6384425
48	(exp health personnel/ or home care personnel/ or (staff or nurse or nurses or Personnel).tw.) and (exp residential care institutions/ or Community Health Services/ or (Community Health Service* or home or homes or Residential Facilit* or living facilit*).tw.)	161771
49	or/43-48	6880984
50	(life adj3 (experience or events or event or transition or transitions or transitional or change or changes)).tw.	112701

51	((self adj3 (management or managing)) or adherence or compliance or coping or resilience).tw.	883991
52	(life adj3 quality).tw.	742298
53	exp life changes/	62090
54	"quality of life"/ or exp life satisfaction/	671001
55	(knowledge or information or education or experience).tw.	7081244
56	information/ or "knowledge (general)"/	32013
57	(need or needs).tw.	3010484
58	or/50-57	10520647
59	42 and 49 and 58	3385
60	exp epilepsy/ or "seizure, epilepsy and convulsion"/	381720
61	(epilep* or seizure* or convulsion*).tw,kw.	560476
62	intellectual disability.af.	123801
63	exp intellectual impairment/	513082
64	((mental or Intellectual or cognitive) and (disabilit* or retardation or Deficiencies or Deficiency or impairment)).tw,kw.	495269
65	(60 or 61) and (62 or 63 or 64)	60995
66	severe myoclonic epilepsy in infancy/	1597
67	(GLUT1 deficiency syndrome or glucose transporter type 1 deficiency syndrome).tw,kw.	526
68	myoclonus epilepsy/	9530
69	lafora.tw,kw.	1472
70	TSC1.tw,kw.	4543
71	TSC2.tw,kw.	5762
72	Landau Kleffner syndrome/	1142
73	Landau Kleffner.tw,kw.	1336

74	Lennox Gastaut syndrome/	4026
75	Lennox Gastaut.tw,kw.	3834
76	(myoclonic epilep* or myoclonic encephalopath*).tw,kw.	7827
77	unverricht-lundborg.tw,kw.	718
78	Sturge Weber syndrome/	3734
79	Sturge Weber.tw,kw.	3797
80	ohtahara syndrome/	342
81	Ohtahara.tw,kw.	597
82	Aicardi syndrome/	544
83	Aicardi.tw,kw.	1794
84	GRIN1.tw,kw.	593
85	or/65-84	92755
86	exp family/	1127632
87	exp "care and caring"/	610418
88	(parent* or mother* or father* or daughter* or son* or sibling* or sister* or brother* or wife* or spouse* or husband* or partner* or meaningful person* or family member* or stepfamil* or next-of-kin or kinship or relatives or caregiver* or carer or carers or dependent* or significant others).tw,kw.	6404461
89	(exp health care personnel/ or (staff or nurse or nurses or Personnel).tw,kw.) and (residential home/ or community care/ or (Community Health Service* or home or homes or Residential Facilit* or living facilit*).tw,kw.)	131498
90	or/86-89	7316473
91	life event/	28437
92	(life adj3 (experience or events or event or transition or transitions or transitional or change or changes)).tw,kw.	113281
93	exp "quality of life"/	694980

94	(life adj3 quality).tw,kw.	752630
95	((self adj3 (management or managing)) or adherence or compliance or coping or resilience).tw,kw.	895120
96	(knowledge or information or education or experience).tw.	7081244
97	knowledge/ or information/	75083
98	(need or needs).tw,kw.	3012317
99	or/91-98	10510248
100	85 and 90 and 99	3986
101	34 use medall	877
102	59 use psyh	396
103	100 use emczd	2644
104	101 or 102 or 103	3917
105	remove duplicates from 104	2999

The Cochrane Library

Date of search: August 16 2019

Results: Total 129: Cochrane reviews 16, Trials 113

#1	MeSH descriptor: [Epilepsy] explode all trees	2199
#2	((epilep* or seizure* or convulsion*)):ti,ab,kw	12494
#3	MeSH descriptor: [Intellectual Disability] explode all trees	1220
#4	(((mental or Intellectual or cognitive) and (disabilit* or retardation or Deficiencies or Deficiency or impairment))):ti,ab,kw	19206
#5	(#1 or #2) and (#3 OR #4)	708
#6	(Dravet syndrome.):ti,ab,kw	67

#7	((GLUT1 deficiency syndrome or glucose transporter type 1 deficiency syndrome)):ti,ab,kw	5
#8	MeSH descriptor: [Lafora Disease] explode all trees	0
#9	(Lafora):ti,ab,kw	4
#10	(TSC1 OR TSC2):ti,ab,kw	35
#11	MeSH descriptor: [Landau-Kleffner Syndrome] explode all trees	1
#12	(Landau Kleffner):ti,ab,kw	9
#13	MeSH descriptor: [Lennox Gastaut Syndrome] explode all trees	25
#14	(Lennox Gastaut):ti,ab,kw	240
#15	MeSH descriptor: [Epilepsies, Myoclonic] explode all trees	57
#16	((myoclonic epilep* or myoclonic encephalopath*)):ti,ab,kw	203
#17	[mh "Unverricht-Lundborg Syndrome"]	4
#18	(Unverricht-Lundborg):ti,ab,kw	11
#19	[mh "Sturge-Weber Syndrome"] OR (Sturge-Weber):ti,ab,kw	13
#20	Ohtahara:ti,ab,kw	2
#21	[mh "Aicardi Syndrome"] or Aicardi:ti,ab,kw	2
#22	grin1:ti,ab,kw	3
#23	(Beauchamp et al.: -#22)	1148
#24	[mh "Family"]	8514
#25	[mh "Caregivers"]	1915
#26	(parent* or mother* or father* or daughter* or son* or sibling* or sister* or brother* or wife* or spouse* or husband* or partner* or meaningful person* or family member* or stepfamil* or next-of-kin or kinship or relatives or caregiver* or carer or carers or dependent* or significant others):ti,ab,kw	150207
#27	([mh "Health Personnel"] or (staff or nurse or nurses or Personnel):ti,ab,kw) and ([mh "Residential Facilities"] or [mh "Community	9936

	Health Services"] or (Community Health Service* or home or homes or Residential Facilit* or living facilit*)):ti,ab,kw'	
#28	(Beauchamp et al.: -#27)	157100
#29	[mh "Life Change Events"]	454
#30	(life NEAR/3 (experience or events or event or transition or transitions or transitional or change or changes)):ti,ab,kw	3754
#31	[mh "quality of life"] or (life NEAR/3 quality):ti,ab,kw	97070
#32	((self NEAR/3 (management or managing)) or adherence or compliance or coping or resilience):ti,ab,kw	72635
#33	(knowledge or information or education or experience):ti,ab,kw	171499
#34	(need or needs):ti,ab,kw	79718
#35	(Larsen et al.: -#34)	342487
#36	#23 and #28 and #35	129

CINAHL EBSCO Host

Date of search: August 16 2019

Results: 1604

S1	(MH "Epilepsy+")	14,990
S2	TI ((epilep* or seizure* or convulsion*)) OR AB ((epilep* or seizure* or convulsion*))	26,315
S3	(MH "Intellectual Disability+")	27,651
S4	TI (((mental or Intellectual or cognitive) and (disabilit* or retardation or Deficiencies or Deficiency or impairment))) OR AB (((mental or Intellectual or cognitive) and (disabilit* or retardation or Deficiencies or Deficiency or impairment)))	51,934
S5	S1 OR S2	29,549
S6	S3 OR S4	70,017

S7	S5 AND S6	2,018
S8	(MH "Dravet Syndrome")	73
S9	TI Dravet syndrome OR AB Dravet syndrome	191
S10	TI ((GLUT1 deficiency syndrome or glucose transporter type 1 deficiency syndrome)) OR AB ((GLUT1 deficiency syndrome or glucose transporter type 1 deficiency syndrome))	43
S11	TI lafora OR AB lafora	48
S12	TI (TSC1 or TSC2) OR AB (TSC1 or TSC2)	196
S13	(MH "Landau-Kleffner Syndrome")	16
S14	TI Landau Kleffner OR AB Landau Kleffner	52
S15	(MH "Lennox-Gastaut Syndrome")	101
S16	TI lennox-gastaut OR AB Lennox Gastaut	173
S17	(MH "Epilepsies, Myoclonic+")	234
S18	TI ((myoclonic epilep* or myoclonic encephalopath*)) OR AB ((myoclonic epilep* or myoclonic encephalopath*))	386
S19	TI Unverricht-Lundborg OR AB Unverricht-Lundborg	24
S20	(MH "Sturge-Weber Syndrome")	150
S21	TI Sturge-Weber OR AB Sturge-Weber	203
S22	TI Ohtahara OR AB Ohtahara	35
S23	(MH "Aicardi Syndrome")	17
S24	TI aicardi OR AB Aicardi	92
S25	TI GRIN1 OR AB GRIN1	15
S26	S2 OR S7 OR S8 OR S9 OR S10 OR S11 OR S12 OR S13 OR S14 OR S15 OR S16 OR S17 OR S18 OR S19 OR S20 OR S21 OR S22 OR S23 OR S24 OR S25	27,102
S27	(MH "Family+")	199,344
S28	(MH "Caregivers")	30,717

S29	TI ((parent* or mother* or father* or daughter* or son* or sibling* or sister* or brother* or wife* or spouse* or husband* or partner* or meaningful person* or family member* or stepfamil* or next-of-kin or kinship or relatives or caregiver* or carer or carers or dependent* or significant others)) OR AB ((parent* or mother* or father* or daughter* or son* or sibling* or sister* or brother* or wife* or spouse* or husband* or partner* or meaningful person* or family member* or stepfamil* or next-of-kin or kinship or relatives or caregiver* or carer or carers or dependent* or significant others))	531,908
S30	(MH "Health Personnel+")	492,168
S31	TI ((staff or nurse or nurses or Personnel)) OR AB ((staff or nurse or nurses or Personnel))	371,005
S32	S30 OR S31	736,275
S33	(MH "Residential Facilities+")	28,516
S34	(MH "Community Health Services+")	375,178
S35	TI ((Community Health Service* or home or homes or Residential Facilit* or living facilit*)) OR AB ((Community Health Service* or home or homes or Residential Facilit* or living facilit*))	130,741
S36	S33 OR S34 OR S35	481,746
S37	S32 AND S36	102,311
S38	S27 OR S28 OR S29 OR S37	707,118
S39	(MH "Life Change Events+")	43,088
S40	TI ((life N3 (experience or events or event or transition or transitions or transitional or change or changes))) OR AB ((life N3 (experience or events or event or transition or transitions or transitional or change or changes)))	18,865
S41	(MH "Quality of Life+")	103,214
S42	TI (quality N3 life) OR AB (quality N3 life)	105,563
S43	TI (((self N3 (management or managing)) or adherence or compliance or coping or resilience)) OR AB (((self N3 (management or managing)) or adherence or compliance or coping or resilience))	119,585

S44	TI ((knowledge or information or education or experience)) OR AB ((knowledge or information or education or experience))	805,869
S45	(MH "Knowledge+")	56,854
S46	(MH "Education+")	798,960
S47	TI (need or needs) OR AB (need or needs)	365,982
S48	S39 OR S40 OR S41 OR S42 OR S43 OR S44 OR S45 OR S46 OR S47	1,729,191
S49	S26 AND S38 AND S48	1,604
