# Supplementary material for: Rare, epilepsy-related disorder including intellectual disability – A scoping review of caregivers’ identified information needs
Source: J Intellect Disabil. 2021 May 17;26(3):704–17. doi: 10.1177/17446295211002348 (PMC9442773; doi:10.1177/17446295211002348)
Supplement: Supplemental Material, sj-pdf-2-jld-10.1177_17446295211002348 - Rare, epilepsy-related disorder including intellectual disability – A scoping review of caregivers’ identified information needs [file sj-pdf-2-jld-10.1177_17446295211002348.pdf]

Appendix 2. Details of the articles

Author; year; country	Title	Informants; number of participants (N)	Study aim	Disease; category of seizures; intellectual level; age	Design; method	Information needs identified for formal/informal caregivers
**Baca, Barry and Berg (2018, USA)**	The epilepsy transition care gap in young adults with childhood onset epilepsy.	Informal caregivers (parent proxy or patients); N=308	To assess the extent to which epilepsy transition discussions occurred (prior to the patients' 18th birthday) in the transition to adulthood	Epilepsy/ epilepsy and ID<br><br>Category of seizures not specified<br><br>14,6 % had IQ<70<br><br>Patients aged 18 years or older	Quantitative; longitudinal study;<br><br>Structured interviews and telephone interviews	**Formal caregivers:**<br><br>-A need for professionals to initiate a transition discussion between the patient, family/local caregiver and neurologist

						/medical formal caregivers in transition to adulthood.<br><br>- The need to identify barriers to successful delivery of effective information in transition is critical to build effective future care models.
**Bar, Ghobeira, Azzi, Ville, Riquet, Touraine, Chemaly and Nabbout (2019, France)**	Experience of follow-up, quality of life, and transition from paediatric to adult healthcare of patients with tuberous sclerosis complex.	Informal caregivers (family members and	To explore the experiences with medical follo	Tuberous Sclerosis Complex and epilepsy	Quantitative;<br><br>Multicentre	**Formal caregivers:**<br><br>-A need to optim

	patients); N= 60	w-up and transition	(debut < 16) Epileptic spasms or focal seizures in 98 % of patients Approx. 80 % had cognitive impairment Patients aged 18 years or older	survey	ise transition programs to increase continuity of information between professionals particularly for patients with cognitive and psychiatric impairment. - A need for information related to the risk of developing symptoms from benig

						n tumours in organs (cortex, the heart, the lunges, the kidneys the skin etc.) that may need medical treatment.
**Bellon, Pfeiffer, Maurici (2014, Australia)**	Choice and control: How involved are people with epilepsy and their families in the management of their epilepsy? Results from an Australian survey in the disability sector.	Informal caregivers (family caregivers and patients); N=171 and N=118 respectively	To explore the involvement in developing an epilepsy management plan	Epilepsy and ID<br><br>Category of seizures not specified<br><br>18 % had various degrees of ID<br><br>Patients	Quantitative; Multicentre online survey	**Informal caregivers:**<br><br>- Family caregivers and patients need to be involved in writing epilepsy management plans in order to

				aged 18 years or older		ensure patient-centred care.
**Both, Holt, Mous, Patist, Rietman, Dieleman, Hoopen, Vergeer, de Wit, de Heus, Moll, van Eeghen (2018, The Netherlands)**	Tuberous sclerosis complex: Concerns and needs of patients and parents from the transitional period to adulthood.	Informal caregivers (parents and patients); N=12 and N=16 respectively	To explore the concerns and care needs in the transition	Tuberous Sclerosis Complex<br><br>Category of seizures not specified<br><br>32,1% (9 patients) had mild ID (IQ: 55-70)/<br><br>17,9% (5 patients) had moderate ID	Qualitative; Semi-structured interviews	**Formal caregivers:**<br><br>-The need for professional caregivers that includes the family caregivers and the patient’s comprehensive needs.<br><br>-The need for patient-centred, multi

(IQ: 40 – 55)/ 7,1% (2 patients) had severe ID (IQ: 20 – 40)<br><br>Patients aged 18 years or older	disciplinary information exchange in transitions to adulthood.<br><br>-The need for well-informed professionals concerning possibility of seizures, side-effects of medication, tumour growth, TSC-associated neuropsychiatric disorders (TAND).

						**Informal caregivers:**<br>The need for peer-support groups for parents.
**Buelow, Mc Nelis, Shore, Austin (2006, UK)**	Stressors of parents of children with epilepsy and intellectual disability.	Informal caregivers (parents); N=20	To explore sources of parental stress	Epilepsy and mild ID<br>Category of seizures not specified<br>All participants had mild ID (IQ = 55-75)<br>Patients aged	Qualitative; Semi-structured interviews	**Formal caregivers:**<br>-The need for interaction between school and parents concerning issues like communication, transitions, child safety, and social issues.

				9-16 years		-The need for information/skills concerning behavioural problems, and the handling of seizures.<br><br>**Formal and informal caregivers:**<br><br>-The need for sufficient information concerning the child`s diagnosis/treatment.

**Deepak,**<br>**Obe,**<br>**Attavar (2012, UK)**	Administration of emergency antiepileptics in staffed care homes for people with intellectual disabilities in High Wycombe: survey of staff training status	Formal caregivers (staff and managers of nursing homes);<br>N= not specified	To determine the training status and -need of staff in nursing homes regarding emergency antiepileptic medication	Epilepsy and ID<br>Category of seizures not specified<br>ID level not specified<br>Patients' age not specified	Quantitative; Survey and telephone interviews	**Formal caregivers:**<br>-The need for seizure-stopping training for formal caregivers in nursing homes.<br>-The need for multidisciplinary collaboration and communication between professionals in various levels of care involved in

						managing people with intellectual disabilities and epilepsy to ensure provision of safe and effective care.
**Desnous, Goujon, Bellavoine, Merdariu, Auvin (2011, France)**	Perceptions of fever and fever management practices in parents of children with Dravet syndrome.	Informal caregivers (parents); N=20	To study parents' experiences of managing fever in their child	Dravet syndrome<br><br>Category of seizures not specified<br><br>ID level not specified<br><br>Patients' mean	Quantitative; Survey	**Informal caregivers:**<br><br>-The need for parent guidance related to appropriate handling of fever.<br><br>-The need for addressing parental

				age 4,7 years		anxiety regarding fever.
**Gordon, Valiengo, Proenca¸ Kurcgant, Jorge, Castro, Marchetti (2014, Brazil)**	Comorbid epilepsy and psychogenic non-epileptic seizures: How well do patients and caregivers distinguish between the two.	Informal caregivers (family caregivers and patients); N=25 and<br><br>N= 24 respectively	To explore how patients and informal caregivers distinguish between epilepsy and psychogenic non-epileptic seizures (PNES)	Epilepsy and ID<br><br>Generalized seizures in 16, 7 %/ Seizure activity in focal-temporal lobe in 66, 7 %/<br><br>Seizure activity in focal-non temporal lobe in 4, 2 %/<br><br>Nonclassified in 12,3 % and	Qualitative; Interviews and video-electroencephalography (VEEG) containing the patients' epileptic seizures and PNES.	**Informal caregivers:**<br><br>-The need for (parental) information/education in order to be able to distinguish between PNES and an epileptic seizure and thus treat the seizures properly.

				PNES in<br><br>100 %<br><br>25% (6 patients) had mild ID / 20,8 % (5 patients) had moderate ID<br><br>Patients aged 18 years or older		
**Jensen, Liljenquist, Bocell, Gammaitoni, Aron, Galer, Amtmann (2017, USA)**	Life impact of caregiving for severe childhood epilepsy: Results of expert panels and caregiver focus groups.	Mix: informal caregivers<br><br>(parents); N=19 / formal caregivers (professional caregivers	To determine what domains to include when measuring caregiver impact	Dravet syndrome or Lennox Gastaut syndrome<br><br>Category of seizures not	Qualitative;<br><br>Interviews and focus group interviews	**Formal caregivers:**<br><br>- The need for trained and skilled formal caregivers in respite

		and expert panel); N=not specified		specified<br><br>ID level not specified<br><br>Patients aged 18 years or older		care with patient-centred information provided by the parents/family caregivers<br><br>-The need for a measure that asses s all of the relevant domains necessary to consider the informal caregiver burden.
**Kehyayan & Hirdes (articles: 2018 & 2019, Canada)**	2018: Profile of persons with epilepsy receiving home care services.	Informal	To descri	Epilepsy	Quantitativ	**Informal**

	2019: Care needs and health care burden of persons with epilepsy receiving home care services.	caregivers (family members and patients); N=5665	be and understand the needs of patients in home care	and ID<br><br>Category of seizures not specified<br><br>27,2 % had intact cognition/<br>47,7 % had mild impairment/<br>11,6 % had moderate impairment/<br>13,4 % had severe impairment<br><br>Patients aged	e; Survey	**caregivers:**<br><br>-The need for information and skills in caring for a family member with epilepsy. This includes the handling of seizures, medication, nutrition, the prevention of injury from falling in a seizure, the training of ADL functions and how

				60 years or older		to handle psychological and/or behavioural difficulties.<br><br>-The need for information regarding how to cope with stress for family caregivers.
**McGrother, Bhaumik, Thorp, Hauck, Branford, Watson (2006, UK)**	Epilepsy in adults with intellectual disabilities: Prevalence, associations and service implications.	Informal caregivers (family caregivers); N=620	To determine the prevalence of epilepsy and associated difficulties in	Epilepsy and ID<br><br>Category of seizures not specified	Mixed methods;<br><br>Medical patient register and interviews	**Formal caregivers:**<br><br>-The need for multidisciplinary collaboration and informatio

			adults with ID	All participant had moderate to severe ID (IQ<55)<br><br>Patients aged 18 and older		n exchange (social services, educational agencies, health professionals, family caregivers, and the voluntary sector).<br><br>-The need for specialists with knowledge related to epilepsy, associated difficulties and ID.
**Nolan, Camfield and Camfield (2008, Canada)**	Coping With a Child With Dravet Syndrome: Insights From Families	Informal caregi	To describe	Dravet	Qualitative;	**Formal**

vers (parents); N=24	potential coping strategies to improve the daily life of parents	syndrome<br><br>Category of seizures not specified<br><br>ID level not specified<br><br>Patients aged 24 or younger	Interviews	**caregivers:**<br><br>-The need for an emergency department protocol for the handling of prolonged seizures to support the various professional caregivers involved in the treatment of the child.<br><br>-The need for teachers to call parents, not asking siblin

gs for aid in case of seizures occurring at school to lessen the burden on siblings

-The need for strategies/actions to minimize pain in children who experience repeated blood sampling.

Informal caregivers:

-The need for emergency routi

nes to ensure a safe treatment of the child`s seizures.

-The need to reduce the burden of care for the parents by learning ways to find practical solutions to every-day health challenges and experiencing emotional support through

						participation in peer-support groups.
**Schultz (2013, UK)**	Parental experiences transitioning their adolescent with epilepsy and cognitive impairments to adult health care	Informal caregivers (parents); N=7	To describe parental transition experiences	Epilepsy and ID<br><br>Category of seizures not specified<br><br>All participants had severe to profound ID (IQ <40)<br><br>Patients aged 18 and older	Qualitative; Interviews	**Formal caregivers**<br><br>-The need to develop transition programme for adolescents with cognitive impairments in particular<br><br>**Formal and informal caregivers:**<br><br>-The need for easily accessible and easily

							understandable information about the diagnosis and the safe handling of seizures.<br><br>**Informal caregivers:**<br><br>-The need for information on how to navigate in the health care and social services.<br><br>-The need for information from

						peers.
**Thomsen, Linehan, Glynn, Kerr (2013 & 2014, UK and Ireland)**	2013: A qualitative study of caregivers ' and professionals' views on the management of people with intellectual disability and epilepsy: A neglected population.<br><br>2014:<br><br>Caring for a family member with intellectual disability and epilepsy: Practical, social and emotional perspectives	2013:<br><br>Mix:<br><br>Informal caregivers (family caregivers); N= 38/ formal caregivers (professionals and<br><br>paid caregivers)<br><br>N=59 and<br><br>N=16 respectively<br><br>2014:<br><br>Informal caregivers (family members); N=48	2013: To study the impact of epilepsy and evaluate the available treatment<br><br>2014: To examine the impact on caregivers	Epilepsy and ID<br><br>Category of seizures not specified<br><br>ID level not specified<br><br>Patients aged<br><br>17 or younger<br><br>38,1 %<br><br>(24 patients)/<br><br>patients aged 18 years or older	Quantitative; International study online survey	**Formal caregivers:**<br><br>-The need for patient-centred information (medical and personal history) to meet the care demand in a safe way.<br><br>-The need for training in administering (acute) medications.<br><br>**Informal**

12,7 %<br><br>(8 patients)	**caregivers**:<br><br>-The need for optimal medical information (including routine medicines) as well as to reduce concerns (psychological stress) during medical consultations.<br><br>-The need for optimal collaboration of services for the profe

						ssionals involved, especially in times of transitions.
**Vallenga, Grypdonck Tan, Lendemeijer-and Boon (2007, the Netherlands)**	Improving decision-making in caring for people with epilepsy and intellectual disability: an action research project.	Mix:<br><br>Informal caregivers (family members) / formal caregivers (professional caregivers);<br><br>N: not specified	To improve decision-making regarding risks	Epilepsy and ID<br><br>Category of seizures not specified<br><br>ID level not specified<br><br>Patients' age not	Qualitative; Interviews and observations	**Formal caregivers:**<br><br>- The need for information and skills in risk management associated with the persons` epilepsy and overall functi

					specif ied		oning . .<br><br>-The need for incre ased dialog ue with the patie nt and the famil y mem bers as a preco nditio n for share d decisi on-maki ng.<br><br>- The need for a more syste matic appro ach amon g the profe ssion al caregi vers, orient ed to indivi dual client

						’s needs, capacity and the severity of the condition.

ID = intellectual disability, PNES = psychogenic non-epileptic seizures
